# Design and Evaluation of Losartan Potassium Effervescent Floating Matrix Tablets: In Vivo X-ray Imaging and Pharmacokinetic Studies in Albino Rabbits

**DOI:** 10.3390/polym13203476

**Published:** 2021-10-10

**Authors:** Mohamed Rahamathulla, Srinivasan Saisivam, Abdullah Alshetaili, Umme Hani, Hosahalli Veerabhadrappa Gangadharappa, Sultan Alshehri, Mohammed M. Ghoneim, Faiyaz Shakeel

**Affiliations:** 1 Department of Pharmaceutics, College of Pharmacy, King Khalid University, Abha 61421, Saudi Arabia; ummehaniahmed@gmail.com; 2 Department of Pharmaceutics, N.R. Vikaria Institute of Pharmacy, Junegad 362001, Gujrat, India; saisivams@yahoo.com; 3 Department of Pharmaceutics, College of Pharmacy, Prince Sattam Bin Abdulaziz University, Al-Kharj 11942, Saudi Arabia; a.alshetaili@psau.edu.sa; 4 Department of Pharmaceutics, JSS College of Pharmacy, JSS Academy of Higher Education and Research, Mysuru 570015, Karnataka, India; gangujss@gmail.com; 5 Department of Pharmaceutics, College of Pharmacy, King Saud University, Riyadh 11451, Saudi Arabia; salshehri1@ksu.edu.sa (S.A.); faiyazs@fastmail.fm (F.S.); 6 Department of Pharmacy Practice, College of Pharmacy, AlMaarefa University, Ad Diriyah 13713, Saudi Arabia; mghoneim@mcst.edu.sa

**Keywords:** losartan potassium, floating tablets, X-ray imaging, in vivo pharmacokinetic studies

## Abstract

Losartan potassium (LP) is an angiotensin receptor blocker used to treat hypertension. At higher pH, it shows poor aqueous solubility, which leads to poor bioavailability and lowers its therapeutic effectiveness. The main aim of this research was to develop a direct compressed effervescent floating matrix tablet (EFMT) of LP using hydroxyl propyl methylcellulose 90SH 15,000 (HPMC-90SH 15,000), karaya gum (KG), and an effervescent agent, such as sodium bicarbonate (SB). Therefore, an EFMT has been developed to prolong the stomach residence time (GRT) of a drug to several hours and improve its bioavailability in the stomach region. The blended powder was evaluated for pre-compression characteristics, followed by post-compression characteristics, in vitro floating, water uptake studies, and in vitro studies. The optimized formulation of EFMT was investigated for in vivo buoyancy by X-ray imaging and pharmacokinetic studies in Albino rabbits. The results revealed that the parameters of pre- and post-compression were within the USP limits. All tablets showed good floating capabilities (short floating lag time <1 min and floated for >24 h), good swelling characteristics, and controlled release for over 24 h. The Fourier-transform infrared (FTIR) and differential scanning calorimetry (DSC) spectra showed drug–polymer compatibility. The optimized formulation F3 (HPMC-90SH 15,000-KG) exhibited non-Fickian diffusion and showed 100% drug release at the end of 24 h. In addition, with the optimized formulation F3, we observed that the EFMT floated continuously in the rabbit’s stomach area; thus, the GRT could be extended to more than 12 h. The pharmacokinetic profiling in Albino rabbits revealed that the relative bioavailability of the optimized LP-EFMT was enhanced compared to an oral solution of LP. We conclude that this a potential method for improving the oral bioavailability of LP to treat hypertension effectively.

## 1. Introduction

In the drug delivery system, the oral route has been the most popular and widely used route of administration. Due to the irregular and changing environment in the gastrointestinal tract (GIT), the traditional per oral dosage forms cannot provide prolonged effective plasma drug concentration and bioavailability. This is due to several physiological difficulties/limitations, such as gastric emptying (GE), stomach pH, gastric motility (GM), gastric residence time (GRT), and so on; this can be resolved by formulating a suitable dosage form [1]. In recent years, controlled-release formulations of various types have been designed to enhance the bioavailability and release of drugs from the system in a controlled manner that are reliable, predictable, and improve patient compliance [1,2]. Gastroretentive drug delivery systems (GRDDS) are unique technologies that have been developed to bypass these limitations [2]. By avoiding the first-pass effect, such methods enhance bioavailability and absorption. The therapeutic level is kept constant through continuous drug release. This can also increase patient compliance by lowering the frequency of dose; drugs with shorter half-lives can produce a significantly better therapeutic effects. One of the most prevalent types of GRDDS is the floating/pulsatile drug delivery system (FDDS/PDDS), which increases the drug absorption by extending the GRT within the upper GIT (effervescent or non-effervescent) [3,4,5,6].

Several approaches/mechanisms have been attempted to attain controlled GRT in GRDDS, such as flotation [7], super-porous hydrogels [8], mucoadhesion [9], swellable and expansion systems [10], ion exchange resins [11], bioadhesive systems [12], low-density systems [13], erosion systems [14], modified shape systems [15], in situ gelling systems [16], and raft-forming systems [16,17]. Losartan potassium (LP) is the primary member of the new category of non-peptide angiotensin 2 receptor antagonists [18]. It significantly lowers the blood pressure through blocking the rennin–angiotensin pathway by reducing the angiotensin II receptor site. After oral ingestion, LP is quickly absorbed by the GIT. It has a short biological half-life of 2 h and its bioavailability is just 32% [19]. It undergoes a quick first-pass metabolism [18,19]. It was therefore chosen as the model drug for the FDDS. In our previous study, we developed an FDDS of LP using a combination of polymers such as hydroxyl propyl methyl cellulose-K4M (HPMC-K4M) and karaya gum (KG) and evaluated the system for in vitro drug release and X-ray imaging [19].

However, pharmacokinetic studies of the LP FDDS were not performed in our previous work. Therefore, the key goal of this study was to develop an effervescent floating matrix tablet of LP with hydrophilic polymers such as HPMC-90SH 15,000 and KG in order to evaluate its in vitro drug release profile, perform X-ray imaging, and measure its pharmacokinetic parameters. KG is made from the dried exudates of the *Sterculia urens* plant, which belongs to the Sterculiaceae family [20]. In the developed FDDS, the feasibility of natural KG was exploited to control the rate of drug release, and the effects of polymers and various additives on drug release were studied. The drug was blended appropriately with various amounts of polymers and other additives, and pre-compression parameters (micromeritic characteristics) were studied before it was compressed into tablets. The floating matrix tablet formulations developed were assessed for post-compression characteristics (thickness, diameter, hardness, friability, drug content uniformity, and weight uniformity test), subjected to water uptake studies, in vitro floating and in vitro drug release studies, and stability studies, and evaluated for the kinetics of drug release and its mechanisms. The in vivo -ray imaging and pharmacokinetic studies were carried out in Albino rabbits.

## 2. Materials and Methods

### 2.1. Materials

LP and HPMC-90SH 15,000 were received as kind gift samples from Adwya Laboratory (Marsa, Tunisia). Lactose was received as a kind gift sample from Shin-Etsu Chem. (Paris, France). Microcrystalline cellulose (MCC) was obtained as a kind gift sample from BDH Chemicals Ltd. (Brussels, Belgium). Magnesium stearate, sodium bicarbonate, and KG were bought from the Reckitt-Benckier Healthcare (Berkshire, UK). All of the other chemicals in this investigation were of analytical reagent grade.

### 2.2. Formulation of Effervescent Floating LP Tablets

For each formulation (F1–F5), a drug and polymer ratio of 1:3 (LP: KG + HPMC-90SH) and other ingredients were mixed for 10 min, to obtain a homogenous mixture. KG and HPMC-90SH served as a release retardant and swellable polymer, SB served as an effervescent agent, and lactose and magnesium stearate as a diluent and lubricant, respectively, as shown in Table 1. Pre-compression characteristics (micromeritic characteristics), such as tapped density (TD), bulk density (BD), Carr’s index (CI), Hauser’s ratio (HR), and angle of repose (AOR), were evaluated for all formulations prior to compression. A 12-station rotary tablet punching machine was used to compress the powder mixture into tablets using 10 mm flat punches (Mini-Press II, Shiv Pharma Engineers, Mumbai, India) [21].

### 2.3. Post-Compression Parameter Evaluation

For the various post-compression characteristics stated in the US Pharmacopeia 2011, the prepared floating LP tablets were evaluated. The hardness, thickness, and diameter of the tablet were determined by the Erweka hardness tester (TBH-125, Darmstadt, Germany). The friability was assessed using the Friability Pharma testing device (PTF-20E), for four min at 25 rpm. The drug content uniformity (DCU) was assessed using a UV spectrophotometer at 205 nm [19] and uniformity weight by a digital balance.

### 2.4. Characterization of the Optimized Formulation

To perform the drug–excipient interaction studies, and to assess the drug’s integrity and compatibility in the formulation, Fourier-transform infrared (FTIR), differential scanning calorimetry (DSC), and surface morphology of tablets were performed with a scanning electron microscope (SEM) [22,23].

FTIR spectroscopy was used to examine the pure LP and its optimized formulation using the potassium bromide pellet technique. In a mortar, 0.1–2% sample was ground with KBr to obtain a fine powder; it was then transferred to a hydraulic press and a small transparent pellet (disc) was produced after a couple of seconds at 10,000 Psi of pressure. The samples were scanned in Bruker optics with a wavenumber from 4000 to 500 cm^−1^ (Tensor 27, Germany).

SEM images were taken before and after dissolution to investigate or observe the morphology of the optimized floating formulation. After hypothesizing the mechanism of drug release and flotation, these two scans were examined for the morphological characteristics. After dissolution, the tablet was removed from the dissolution apparatus, the surplus water on the tablet was removed with filter paper, and the tablet was dried for 24 h at ambient temperature. The SEM images of gold–palladium samples were captured at a magnification of 500× at a 0.98 torr press and accelerating current of 20 kV.

The DSC was performed on pure LP and the optimized formulation (F3). The calorimetric measurements were carried out using a blank cell as a control (high-purity alpha-alumina disc). In a nitrogen environment, the dynamic spectra were recorded at a heating rate of 10 °C min^−1^, and the heat was quantified as J/kcal (Shimadzu DSC-60A. Japan).

### 2.5. In Vitro Floating Behavior Studies (Buoyancy Test)

The floating lag time (FLT) and total floating time (TFT) were used to distinguish the in vitro floating behavior. The test was carried out in a water bath at 37 ± 0.5 °C with a 200 mL glass beaker containing one hundred milliliters of a pH 1.2 HCl solution (dissolution media) [21]. FLT and TFT were used to measure the time required for the tablet to reach the surface of the dissolution medium, i.e., FLT, and the extent to which it remained buoyant/floating on the medium, i.e., TFT.

### 2.6. Swelling Index (SI) or Water Uptake (WU) Studies

The ability of the tablets (polymers) to absorb water and swell was used to determine their WU. Throughout the investigation, the tablet’s SI was achieved with a USP dissolution apparatus type-1 (Erweka DT-600—Basket type) in nine hundred milliliters of HCl solution pH 1.2, at 100 rpm at 37 ± 0.5 °C [24]. The tablets were retrieved after 8 h from the basket, the surplus water was removed with the help of filter paper, and the tablets were eventually weighed. The SI was determined using Equation (1):

(1) $$\mathrm{SI}\;\left(\%\right)=\frac{\mathrm{Swollen}\;\mathrm{tablet}\;\mathrm{weight}-\mathrm{Original}\;\mathrm{tablet}\;\mathrm{weight}}{\mathrm{Original}\;\mathrm{tablet}\;\mathrm{weight}}\times 100$$

### 2.7. In Vitro Drug Dissolution/Release Studies

The in vitro drug release of LP from floating tablets was evaluated by the Erweka dissolution type-II (Paddle-DT-600) apparatus using nine hundred milliliters of 0.1N HCl at 37 ± 0.5°C at 100 rpm. For the next 24 h, 5 mL of sample solution was removed from the dissolution apparatus at specified time intervals (1, 2, 4, 6, 8, 10, and up to 24 h). To maintain the sink condition, the same volume of fresh dissolution medium was added to the dissolution apparatus at each time interval. The sample was filtered using a membrane filter (0.45 μm) and suitably diluted with 0.1 N HCl before measuring the absorbance. The samples were spectrophotometrically (Analytikajena, Specord-40) analyzed at 205 nm [24]. PCP dissolution software (v 2.08, Pune, India) was used to fit the release kinetics into various mathematical models.

### 2.8. Drug Release Kinetics

To better understand the mechanism and kinetics of drug release from the effervescent LP tablets, various mathematical models were applied. The one that best fits the experimental results is the most appropriate. Applying dissolution software (PCP-Disso, V 2.08, Pune, India), the release kinetics were determined by determining the best fit Higuchi matrix, zero-order, Korsmeyer–Pappas, Hixson–Crowell, and first-order values [25,26,27].

### 2.9. In Vivo Studies

In vivo studies were performed in male Albino rabbits (2.1–2.6 kg) after approval by the “Institutional Animal Ethics Committee (IAEC) of East-West College of Pharmacy, Bangalore, India (Reg. No: 261/2009/CPCSEA)”. The animal use and experimental procedures followed EU directive 2010/63/EU.

#### 2.9.1. Evaluation of Gastric Retention Using X-ray Imaging in Rabbits

An X-ray imaging investigation was performed on the Albino rabbits (n = 6.0) to assess the stomach retention characteristic of the floating tablets. In this study, the drug was substituted with a contrast agent (barium sulfate) to make the tablet visible under an X-ray, and other ingredients were kept constant. Before the experiment, the animals were fasted for 12 h. An X-ray image of a rabbit’s empty stomach was taken, to verify that no radio-opaque materials were present in the stomach region. The optimized tablet (F3) was administered to the rabbit by natural swallowing, followed by 30 mL of water. X-ray photographs were obtained for rabbits in an upright posture at different time intervals during the zero, second, sixth, and twelfth hours using an X-ray machine (Bharath Electronics and Emego-THX 1-125/80 102/THX-1 H, Netherlands). For every X-ray image taken to observe the tablet movements in the stomach region, the space in between the X-ray source and the rabbit (object) was kept constant.

#### 2.9.2. In Vivo Pharmacokinetic Studies of LP Tablets

In this methodology, six rabbits were employed for each group: the first group received the reference standard (pure LP drug solution), while the second group received the optimized effervescent floating matrix tablet of LP (F3). As per the CPCSEA norms, all rabbits that were used for the study were quarantined in an animal house and fasted overnight. They were only permitted access to drinking water. The appropriate dose of pure LP (10 mg) solution was given orally to one group of rabbits through oral gauze. To ensure that the full drug entered the stomach, a few mL of distilled water was additionally delivered using a syringe (without a needle). The optimized EFMT (F3) was administered orally to the second group of rabbits using oral gauze. Blood was taken from the marginal ear vein of the rabbits at regular intervals of time, 0.5 h, 1 h, 2 h, 4 h, 8 h, 12 h, 16 h, and 24 h, using a fine-gauge needle. The blood samples were heparinized and frozen at −80 °C before analysis.

#### 2.9.3. Determination of LP in Rabbit Plasma Samples

The amount of LP in rabbit plasma samples was analyzed using a reversed-phase high-performance liquid chromatography (RP-HPLC) method. An HPLC system coupled with a UV–visible detector (Shimadzu, Japan) was used to analyze LP, utilizing valsartan as an internal standard (IS). The HPLC separation of LP and IS was carried out on an ODS C_18_ column (250 mm × 4.6 mm, particle size 5 μm) maintained at 30 °C. The mobile phase used in this work was composed of phosphate buffer: acetonitrile (75:25, % *v*/*v*, pH 2.8). The mobile phase was delivered at a flow rate of 1.2 mL min^−1^. The detection of LP was performed at 205 nm. The injection volume was 20 µL. The samples were prepared by the protein precipitation method using acetone. Plasma samples (200 µL) were transferred to 2.0 mL centrifuged tubes and 20 µL of IS (100 ng mL^−1^ in acetonitrile) was added. The samples were vortexed for approximately one min and 400 µL of acetone was added. The samples were again gently vortexed for approximately two min and centrifuged at 14,000 rpm for 15 min. The supernatant was carefully taken and evaporated to dryness. The residue was reconstituted with an appropriate quantity of mobile phase and 20 µL was injected into the HPLC system for the analysis.

#### 2.9.4. Pharmacokinetic Parameters

PK summit solutions software (Rock, AR, USA)was used to analyze the pharmacokinetic parameters, such as peak plasma concentration (C_max_), time to reach peak plasma concentration (T_max_), area under curve from time zero to t AUC_0−t_, plasma half-life (t^1/2^), and elimination rate constant (Kel).

### 2.10. Stability Studies

A glass bottle with screw caps was used for packaging the optimized formulation, and twelve-month studies were performed, maintaining the optimized formulation F3 at 40 ± 2 °C and 75 ± 5% relative humidity (RH), 30 ± 2 °C, and 65 ± 5% RH, and 25 ± 2 °C and 60 ± 5% RH. The samples for accelerated storage conditions were removed after 0, 3, 6, and 12 months to verify the changes in drug content and physical appearance, as per the guidelines of the ICH Q1A (R2) [28,29].

### 2.11. Statistical Analysis

The rate of in vitro drug dissolution and various in vivo parameters for LP EFMT were determined by one-way variance analysis (ANOVA) utilizing SPSS software (Version 12, San Diego, CA, USA) [30].

## 3. Results and Discussion

In order to improve the absorption and bioavailability of the LP, it is essential for the dosage form to release the drug to the upper gastric region (stomach). Therefore, an LP gastroretentive formulation was developed and optimized. It is critical to have a lag time (buoyancy in the stomach) in regulated release for chronotherapeutic drug delivery. To control the drug release, the EFMTs were prepared using rate-retarding gel-forming polymers such as KG and HPMC-90SH, along with SB and lactose act as a gas-generating agent and filler, respectively.

### 3.1. Pre-Compression Parameters

The flow and compressibility behavior of the formulations’ (F1–F5) particulate mixtures were assessed using pre-compression parameters. The BD and TD for the mixture were found to be between 0.566 ± 0.23 and 0.612 ± 0.06 g mL^−1^ and between 0.625 ± 0.015 and 0.666 ± 0.014 g mL^−1^, respectively. Despite the diverse polymer compositions (HPMC-90SH, KG, and MCC) in the formulations, the powder combination packing arrangement did not statistically differ (*p* > 0.05). The CI, which measures the stability and possible compression bonds, was determined to be excellent. The angle of repose (AOR) was observed to be between 20.80° ± 0.17 and 23.74° ± 0.61° for the powder mixture, which indicated that it was suitable for compression. The HR was determined to be less than 1.15, indicating the acceptable flow of the powder combination. As shown in Table 2, the findings of these pre-compression parameters were within USP limitations.

### 3.2. Post-Compression Parameters

The matrix tablets showed uniform thickness in the range of 4.00 ± 0.92 to 4.20 ± 0.33 mm. The in vitro LP and drug release in a GRDDS may be affected by the tablet’s hardness. The hardness of the tablet had no or only a slight influence on the drug release profile; however, it was a crucial factor in the tablets’ buoyancy/floating. The rate of penetration of the dissolving media into the tablet was directly influenced by the tablet’s hardness [31]. Depending on the aforesaid observation, the floating matrix tablet’s hardness of LP was adjusted to around 50 N. The LP floating matrix tablet’s hardness was found to be in the range of 49 ± 0.33 to 55 ± 0.24 N. The tablet’s friability varied between 0.76 ± 0.08 and 0.92 ± 0.03%.

All formulations had good mechanical resistance in terms of friability. The weight uniformity and DCU of the LP matrix tablets met USP specifications. Results are shown in Table 3, and all post-compression parameters of the developed LP matrix FT were within the official limits of the US Pharmacopeia 2011.

### 3.3. FTIR Spectra

**Figure 1:**
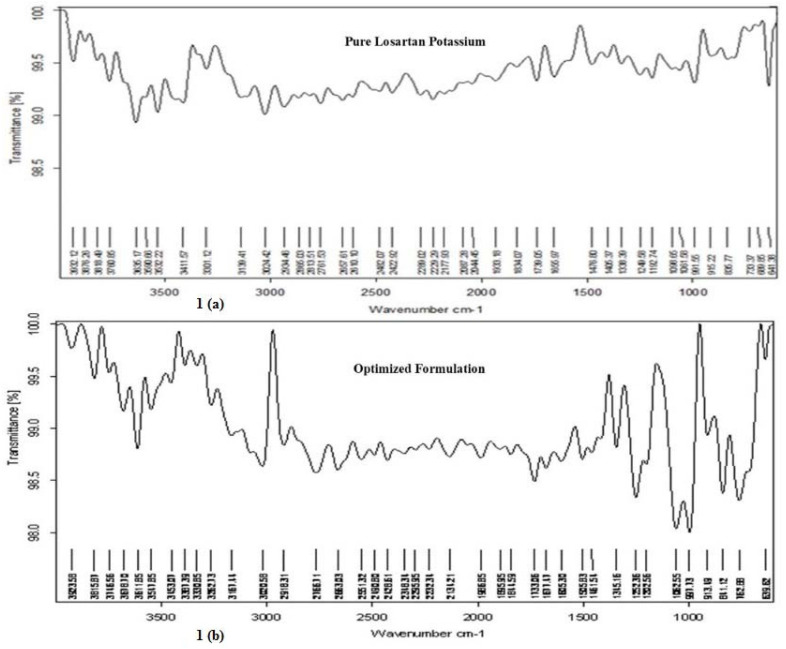
(**a**) FTIR spectra of pure losartan potassium (LP), (**b**) FTIR spectra ofoptimized floating tablet formulation (F3).

The pure LP’s FTIR spectra showed principal peaks at various wavenumbers, such as O–H stretching at 3139 cm^−1^, C–H stretching at 2934 cm^−1^, N=N stretching at 1656 cm^−1^, C=C stretching at 1478 cm^−1^, C–N stretching at 1243 cm^−1^, and C–Cl stretching at 733 cm^−1^, as illustrated in Figure 1. The optimized EFMT formulation’s (F3) spectra showed all the standard peaks found in the pure LP, indicating that the polymers utilized in the EFMT of LP formulations did not cause any significant alterations in the standard peaks. Thus, there was no interaction between the polymer and drug in the EFMTs of LP.

### 3.4. SEM Study

**Figure 2:**
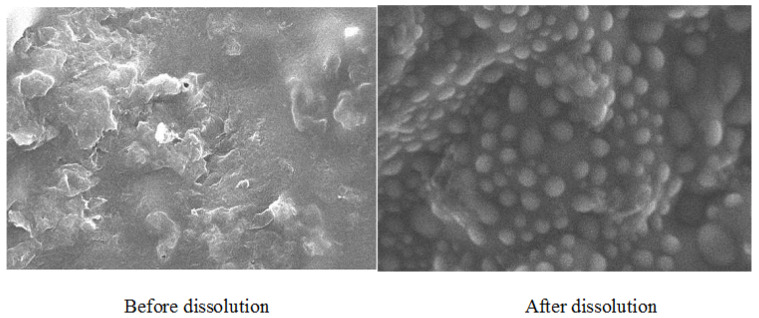
SEM images of an optimized tablet (F3) before and after dissolution.

Before and after dissolution, SEM images of the tablet were taken, as shown in Figure 2. The SEM images clearly showed that the surface of the tablet was rough, with no perforation, troughs, or canals, before the dissolution of the tablet. When the dissolution medium (solvent) came into contact with the tablet, the solvent penetrated the tablet matrix gradually, and then the drug diffused from the matrix. After dissolution studies, the tablet surface morphology showed a significant change due to the formation of pores and surface cracks. The optimized tablet’s (F3) SEM images revealed a web-like network from which the drug diffused to the surrounding material. As a result, it was found that the drug was released from the matrix by a diffusion process (non-Fickian release).

### 3.5. DSC Evaluation

**Figure 3:**
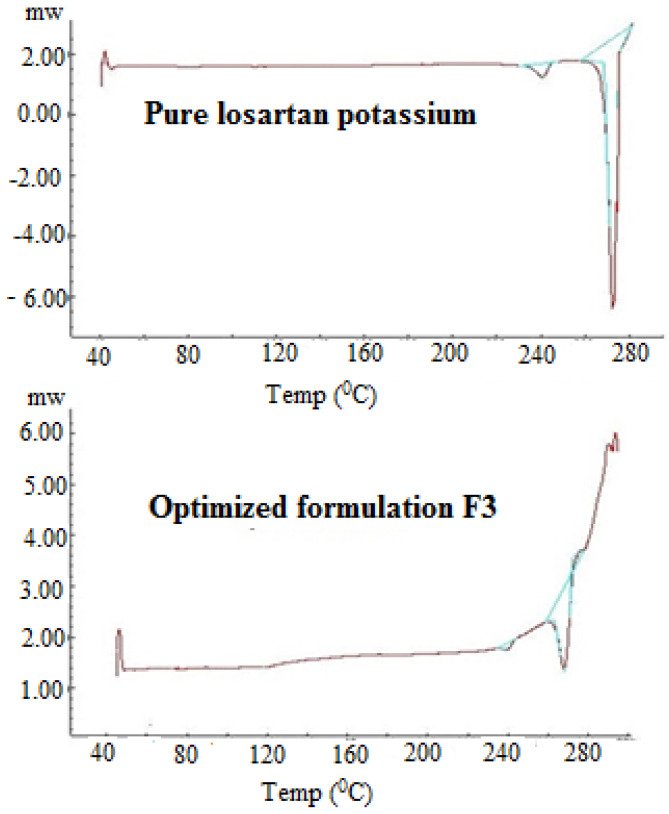
DSC thermograms of pure LP and optimized floating tablet formulation F3.

DSC evaluation is a fast and efficient process for checking drug and excipient compatibility and to obtain the most information on possible interactions. Pure LP and its optimized formulation F3 were subjected to DSC analysis. The pure LP thermogram displayed an endothermal high peak at 272.55 °C, which was its melting point. An endothermic peak of LP was slightly shifted to 273.04 °C in the optimized formulations (F3). In addition, the intensity of LP was significantly reduced in optimized formulation F3, suggesting the entrapment of the drug into the polymers used. Because the melting point of LP was slightly changed in optimized formulation F3, it can be concluded that there was no chemical interactions between the LP and the polymers studied (Figure 3).

### 3.6. In Vitro Buoyancy Studies

**Figure 4:**
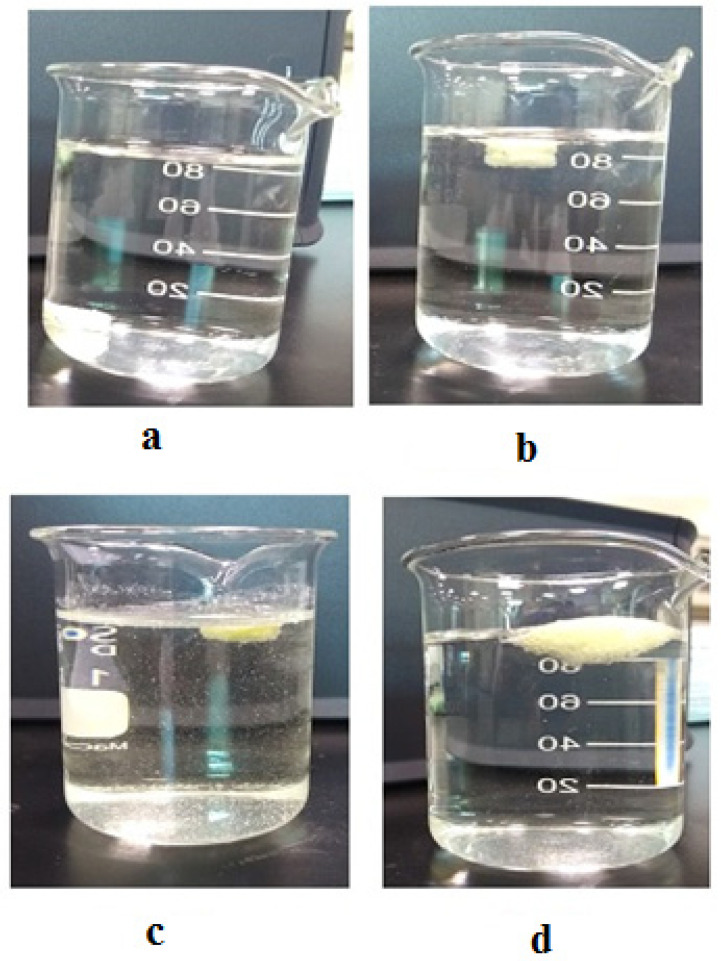
Photographs of tablet (F3) to illustrate floating lag time. (**a**) At zero time, (**b**) At 16 s, (**c**) At 8th h, (**d**) At 12th h.

The LP matrix tablet prepared from the hydrophilic polymers did not display any gastric retention or floating behavior in the stomach. To prolong the GRT in the FDDS, the researchers adopted an effervescent method. Several effervescent agents (gas-generating), such as citric acid, tartaric acid, SB, or calcium carbonate, have been used in the past. In our studies, we selected SB as an effervescent agent; it was a critical component of the effervescent floating matrix tablet. Once the matrix tablet came into contact with the dissolution media (0.1 N HCl), we observed the evolution of CO_2_ (due to the presence of sodium bicarbonate), which was entrapped within the gel generated by the hydration of HPMC-90SH and KG, hence lowering the density of the tablet. The tablet floated when the density fell below 1.0. HPMC-90SH and KG created highly gel-resistant tablets and the entrapped carbon dioxide gas resulted in steady and sustained floating throughout the experiment. Therefore, SB was a key component to attain optimal floatability. The longer gastric residence period for LP in the stomach may lead to greater absorption. In the FLT of tablets, the concentration of SB played a vital role. The floating lag time decreased as the SB concentration increased and vice versa. The optimum lag time and total floating time were seen at 12% of SB. Hence, 12% SB was selected for the study of the LP floating matrix formulation [32]. It was found that the lag time of all formulations was between 16 ± 2 and 57 ± 2 s, and they continued to float for more than 24 h without tablet decay, as shown in Table 4. Photographs illustrating the F3 floating lag time are shown in Figure 4.

### 3.7. Swelling Index Study

**Figure 5:**
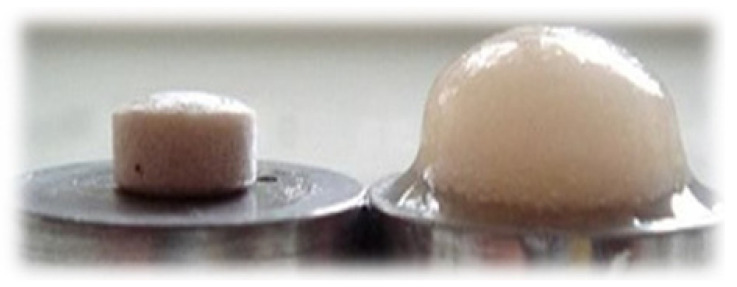
Swelling index of floating matrix tablets of LP (F4).

The swelling index or water uptake studies, or the hydration capacity evaluation, of the formulation demonstrated the ability of the polymer to absorb water and swell when it came into contact with the dissolution medium. The swelling capacity of GR is essential because it affects the floating of tablets, and their kinetic drug release [33]. By the end of 8 h, complete swelling was achieved; thus, for all formulations, the percentage of swelling was measured. F1 and F4 showed the lowest and highest percentage of swelling, i.e., 173.32 ± 4.70 and 640.23 ± 7.70%, respectively, among all formulations. Figure 5 shows the highest swelling of F4. It was noted that the water intake capacity increased significantly and swelled with an increase in the concentrations of HPMC-90SH and KG in the formulation, as reported in Table 4. Drug diffusion depends considerably on the tablet’s water content. This might strongly depend on the water content in the system due to the mobility of the polymer chain. Relaxation of the polymer chain with high water content is achieved by an increase in volume that effectively increases network expansion. This larger water content might also indicate the greater gastric fluid penetration into the tablet, which will result in the faster formation of carbon dioxide gas and thus reduce the FLT [34]. As a result, quicker and higher tablet swelling caused an increase in tablet dimensions, which led to an increasing diffusion pathway, hence lowering or decreasing the rate of diffusion. Thus, initially substantial drug release and later gradually reduced drug release were observed.

### 3.8. In Vitro Drug Release

**Figure 6:**
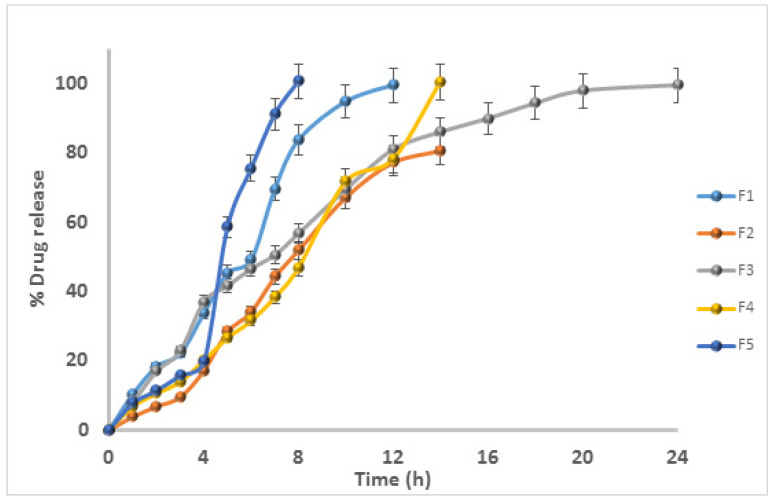
In vitro release profiles of LP floating matrix tablets (F1–F5) (mean ± SD, n = 3.0).

The FDDS can enhance systemic drug bioavailability by extending the GRT of LP, which has better absorption in the stomach region or upper part of the small intestine. The various concentrations of hydrophilic polymers, natural gum, and diluents had a significant effect on the in vitro release of drugs. For all formulations, the in vitro drug release studies were performed in triplicate. Formulations F5 and F1 exhibited around 100% drug release at 10 h and 12 h, respectively. Formulation F1 contained only HPMC-90SH, without KG, while F5 contained only KG, without HPMC-90SH. Formulations F2, F3, and F4 contained blended polymers of HPMC-90SH and KG, with 100% drug release at the 18th, 24th, and 14th h, respectively. Figure 6 shows the in vitro dissolution profile. The results revealed that, if the KG concentration increased, the rate of drug release decreased at a certain concentration [35]. With a further increase in the KG concentration, the rate of drug release increased due to the erosion of the tablet. As the gel barrier thickness increased, the drug required more time to diffuse through it [24], as proven by formulations F2, F3, F4, and F5. The formulations F2, F3, and F4 contained varied proportions of the polymer blend of HPMC-90SH and KG. The results revealed that, in order to control the drug release for 24 h in the system, a single polymer was unsuitable for LP. The blend of polymers used in the study was able to control the rate of drug release for up to 24 h. There were no significant differences in the drug release patterns of the other formulations (*p* > 0.05). Moreover, the rate of drug release of optimized formulation F3 was shown to be significant when compared to the other formulations, F1, F2, F4, and F5 (*p* < 0.05). Most of the formulations analyzed revealed continuous LP release. The drug should be released predictably through an ideal controlled drug delivery system. The system aims to ensure the drug’s safety and efficacy, as well as enhancing patient compliance. The findings indicated that KG could be used to make gastric floating controlled delivery systems. It was clear that nearly all the formulae were successful in maintaining the drug release rate. Moreover, the type and concentration of the polymers investigated affected the rate of drug release. The F3 formulation indicated the continuous release of the drug for up to 24 h. It was chosen as an optimized formulation for in vivo studies.

### 3.9. Drug Release Kinetics

Different models were fitted to provide the in vitro profiles for drug dissolution, and release data were analyzed using the Korsmeyer–Peppas equation (KPE). Release values “k” and “n” were measured using PCP disso software version 2.08 (Pune, India). The exponent for diffusion was between 0.7366 and 1.05645. The correlation coefficients (R^2^) were employed to determine the accuracy of the fitting of the model. For the Korsmeyer–Peppas, Hixson–Crowell, Higuchi, zero-order, and first-order model, the R^2^ values were determined and compared. The formulations F4 and F5 were best fitted in the KP model, the F1 and F2 best fit the Hixson–Crowell model, and F3 best fit the matrix model, as revealed by the coefficients of R^2^ and “k” values. If the “n” value was 0.45 or below in the Korsmeyer–Peppas model, the release mechanism was Fickian diffusion; if “n” was (larger than 0.45 value < one), the mechanism of release was non-Fickian diffusion or anomalous transport. If the “n” value was one, this indicated case-I transport, while an “n” value of more than one indicated that the release mechanism was super case-II transport. This model is useful for analyzing pharmaceutical polymer dosage form releases, when more than one type of release phenomena may be implicated or the release mechanism is unknown [36]. Fick’s rules describing the macroscopic transportation of molecules via a concentration gradient are key to diffusion. The formulations F1, F3, and F4 exhibited non-Fickian release, while F2 and F5 showed super case-II transport. The R^2^, “n”, and “k” values for the tablets, and the drug release mechanisms, are shown in Table 5.

### 3.10. X-ray Imaging

**Figure 7:**
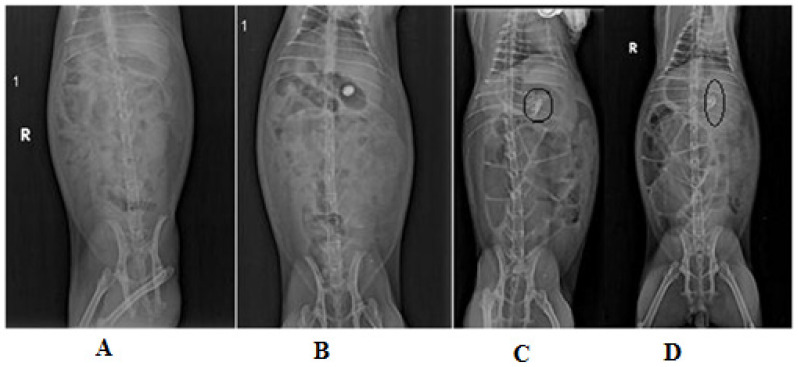
X-ray images of the GIT of a rabbit, showing the movement of an optimized floating tablet (F3) in the stomach (**A**) before administration (empty stomach), (**B**) 2 h after administration, (**C**) at 6 h, and (**D**) after 12 h.

An X-ray radio-opaque marker was utilized in the study to capture instantaneous photographs of an optimized floating matrix tablet (F3) and the GIT background. Even with an inexperienced eye, the transition of the tablet in the GIT was easily detectable. Each image could be read individually due to the organs being highlighted. In rabbits, the optimized floating matrix tablet’s (F3) gastric/stomach retention property was investigated. X-ray photographs were captured at various time intervals after the drug-free formulation was administered to the rabbits (Figure 7).

The X-ray image clearly illustrated the absence of the tablet in the rabbit’s GIT (Figure 7A). Two hours after the oral administration of the tablet, an X-ray image of the stomach was taken and the EFMT was clearly detected in the stomach’s upper portion, as shown in Figure 7B. The X-ray imaging of the tablet at the 6th h and 12th h indicated clearly that the tablet was present in the region of the stomach but had shifted its location in the stomach area, as illustrated in Figure 7C,D, respectively. This demonstrated its gastric retention. This experiment revealed that the optimized EFMT floated continuously in the stomach area of the rabbits, so the gastric retention time could be extended to over 12 h.

### 3.11. In Vivo Pharmacokinetic Studies

**Figure 8:**
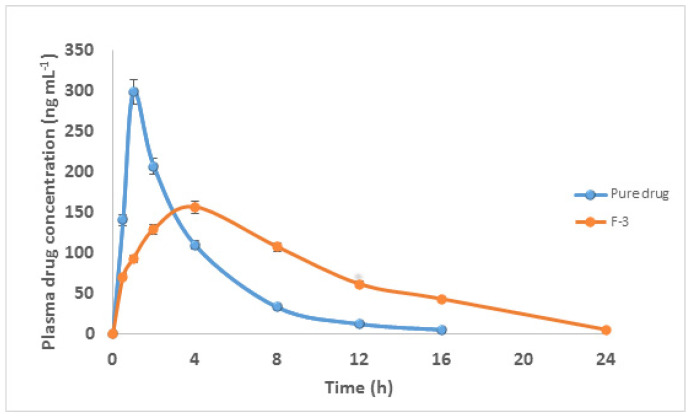
Plasma concentration profiles of pure LP and its optimized formulation F3 following oral administration to rabbits (mean ± SD, n = 6.0).

LP was well separated under the given chromatographic conditions. There were no interference peaks from the endogenous plasma components. For the pharmacokinetic study, the typical chromatogram of blank plasma was compared to that of spiked samples. The LP retention time was 3.56 min. The LP calibration curve was linear over the range of 25–500 ng mL^−1^. On Albino rabbits, in vivo investigations for the pure drug solution and chosen optimal formulations (F3) containing 10 mg of LP were performed. Blood samples were collected at different intervals of time and LP plasma concentrations were analyzed. The plasma concentration–time profile curves of pure LP and an optimized formulation F3 are displayed in Figure 8. As per the data collected, the C_max_ following oral administration was 298.4 ± 12.45 ng mL^−1^ for pure LP and 148.4 ± 15.86 ng mL^−1^ for F3. T_max_ for pure LP was 1.5 h, and for F3, it was 4.1 h. The obtained AUC_0–24_ values were 928.12 ± 51.67 and 1382.40 ± 112.23 ng.h mL^−1^ for pure LP and F3, respectively. The MRT was found to be increased from 4.223 ± 0.07 h for an oral solution to 18.92 ± 0.21 h, respectively, with the sustained-release floating matrix tablet formulations, as shown in Table 6. A statistical investigation using a t-test showed that the oral and optimized formulations differed significantly (*p* < 0.05). Considering the AUC_0–24_, T^1/2^, and Kel, it can be assumed that they exhibited controlled/prolonged drug release.

### 3.12. Stability Studies

Stability studies were performed to evaluate the changes in drug content and physical appearance by raising the temperature and RH. The optimized EFMT of LP (F3) was exposed to stability studies as per the regulation of ICH for 12 months by storing it at 40 °C/75% RH, 30 ± 2 °C/65 ± 5% RH, and 25 °C/60% RH. These samples were evaluated for changes in physical appearance and drug content at regular time intervals. The results for physical appearance and drug content analysis at different temperatures are summarized in Table 7. The physical appearance of optimized formulation F3 was unchanged at all conditions of the stability studies. The drug content of the optimized formulation was obtained in the range of 99.39–100.10%, 98.98–100.10%, and 98.69–100.10% at 25, 30, and 40 °C, respectively. These data showed that the optimized formulation F3 was sufficiently stable under accelerated conditions of temperature and RH.

## 4. Conclusions

HPMC-90SH and natural KG as a swelling and retarding polymer, respectively, with sodium bicarbonate as an effervescent agent, were used to successfully develop a potential controlled-release LP floating tablet. In vitro buoyancy was a better parameter to evaluate for enhancing the absorption of LP. The experiment’s findings showed that the release of the drug from the optimized EFMT (F3) was found to offer optimum buoyancy and controlled drug release, via non-Fickian diffusion. Based on in vivo studies via X-ray imaging in Albino rabbits, the formulation F3 exhibited the floatability of the tablet in the stomach and extended the GRT to around 12 h. Thus, floating tablets might successfully be utilized to regulate the release of LP, which has a narrow absorption window in the upper part of the GIT or stomach. Furthermore, pharmacokinetic investigations showed that, in the orally administered optimized formulation F3, the mean residence time was 19 h and the AUC_0–24_ in plasma was 1382.40 ± 112.23 ng.h mL^−1^, in Albino rabbits, which was significantly higher than the oral solution of the pure LP (*p* < 0.05). These results showed that the optimized F3 formulation may substantially extend the residence time of the drug in vivo in comparison to an LP oral solution and increase its bioavailability through its good gastric floatation capacity and regulated release of the drug into the acidic environment. F3 is therefore a potential formulation of LP to improve its effectiveness and bioavailability. In conclusion, the developed EFMT could be successfully employed for the controlled release of LP.

## Figures and Tables

**Table 1:** Composition of losartan potassium (LP) effervescent floating matrix tablets (F1–F5).

Batch Code	LP	HPMC-90 SH 15,000	KG	MCC	SB	MS	LAC	TOTAL (mg)
F1	50	150	-	15	36	3	46	300
F2	50	125	25	15	36	3	46	300
F3	50	100	50	15	36	3	46	300
F4	50	50	100	15	36	3	46	300
F5	50	-	150	15	36	3	46	300

LP: losartan potassium, KG: karaya gum, MCC: microcrystalline cellulose, SB: sodium bicarbonate, MS: magnesium stearate, LAC: lactose.

**Table 2:** Micrometric properties of LP effervescent floating matrix tablets (F1–F5).

Batch Code	Bulk Density (g mL^−1^) ± SD *	Tapped Density * (g mL^−1^) ± SD	Hauser Ratio	Carr’s Index * (%)	Angle of Repose ± SD *
F1	0.566 ± 0.23	0.625 ± 0.015	1.10	9.44 ± 0.55	21.80 ± 0.29
F2	0.576 ± 0.35	0.625 ± 0.021	1.13	11.65 ± 1.0	22.58 ± 0.34
F3	0.576 ± 0.19	0.666 ± 0.014	1.15	13.5 ± 0.83	23.02 ± 0.73
F4	0.588 ± 0.38	0.652 ± 0.021	1.12	9.81 ± 0.91	23.74 ± 0.61
F5	0.612 ± 0.06	0.666 ± 0.014	1.08	8.10 ± 0.27	20.80 ± 0.17

* Mean ± SD, n = 3.0.

**Table 3:** Post-compression parameters of LP effervescent floating matrix tablets (F1–F5).

Batch Code	Variation of Weight * (%)	Hardness * (N)	Thickness * (mm)	Friability * (%)	Content Uniformity * (%)
F1	2.32 ± 0.19	55 ± 0.24	4.10 ± 0.92	0.76 ± 0.08	100.19 ± 0.14
F2	2.33 ± 0.12	49 ± 0.33	4.20 ± 0.33	0.92 ± 0.03	99.29 ± 0.33
F3	1.66 ± 0.06	53 ± 0.83	4.10 ± 0.89	0.79 ± 0.08	100.17 ± 0.64
F4	1.99 ± 0.02	53 ± 0.68	4.00 ± 0.92	0.88 ± 0.02	99.54 ± 0.12
F5	2.33 ± 0.18	52 ± 0.92	4.20 ± 0.28	0.81 ± 0.07	102.21 ± 0.89

* Mean ± SD, n = 3.0.

**Table 4:** Floating lag time, swelling index, and total floating time of LP effervescent floating matrix tablets (F1–F5).

Batch Code	Floating Lag Time * (s)	Swelling Index * (%)	Total Floating Time (h)
F1	33 ± 3	173.32 ± 4.70	>24
F2	40 ± 3	462.18 ± 5.50	>24
F3	16 ± 2	488.09 ± 4.80	>24
F4	57 ± 2	640.23 ± 7.70	>24
F5	49 ± 1	534.15 ± 6.30	>24

* Mean ± SD, n = 3.0.

**Table 5:** Kinetic treatment of dissolution profile of tablets (values of R^2^, k, and n for tablets) and mechanism of drug release.

Batch Code	Korsmeyer–Peppas		Matrix		Hixson–Crowley		1st-Order		Zero-Order		n	Mechanism of Drug Release	Release Kinetics
	R^2^	k	R^2^	k	R^2^	k	R^2^	k	R^2^	k			
F1	0.989	8.207	0.967	18.319	0.992	10.027	0.991	10.119	0.847	14.88	0.8061	Non-Fickian	Hixson–Crowley
F2	0.997	10.01	0.995	10.018	0.995	10.018	0.990	10.069	0.962	3.834	1.0545	Super case-II transport	Hixson–Crowley
F3	0.995	9.084	0.997	15.761	0.977	10.022	0.988	10.091	0.931	4.329	0.7366	Non-Fickian	Matrix
F4	0.994	6.281	0.927	14.590	0.984	10.022	0.973	10.091	0.978	4.137	0.8597	Non-Fickian	Peppas
F5	0.985	9.127	0.882	26.290	0.874	10.072	0.979	13.159	0.868	6.785	1.0032	case-II transport	Peppas

**Table 6:** In vivo pharmacokinetic data of pure LP and optimized effervescent floating matrix tablet F3 (mean ± SD, n = 6.0).

Parameters	Oral Solution	F3
C_max_ (ng mL^−1^)	298.4 ± 12.45	148.4 ± 15.86
T_max_ (h)	1.5 ± 0.10	4.1 ± 0.35
T_1/2_ (h)	2.38 ± 0.32	3.45 ± 0.25
K_el_ (h)	0.34 ± 0.05	0.27 ± 0.07
AUC_(0-t)_(ng.h mL^−1^)	928.12 ± 51.67	1382.40 ± 112.23
MRT (h)	4.223 ± 0.07	18.92 ± 0.21

**Table 7:** Stability study data of optimized LP tablet F3 (mean ± SD, n = 3.0).

Stability Condition	Sampling Interval (Months)	Physical Appearance	Drug Content (%)
25 ± 2 °C/60 ± 5% RH	0	No change	100.10 ± 0.04
	3	No change	99.91 ± 0.03
	6	No change	99.73 ± 0.02
	12	No change	99.39 ± 0.05
30 ± 2 °C/65 ± 5% RH	0	No change	100.10 ± 0.08
	3	No change	99.84 ± 0.06
	6	No change	99.57 ± 0.04
	12	No change	98.98 ± 0.09
40 ± 2 °C/75 ± 5% RH	0	No change	100.10 ± 0.09
	3	No change	99.38 ± 0.07
	6	No change	98.69 ± 0.06

## Data Availability

The data presented in this study are available on request from the corresponding author.

## References

[1] Singh B.N., Kim K.H. (2000). Floating drug delivery systems: An approach to oral controlled drug delivery via gastric retention. J. Control. Release.

[2] Maroni A., Zema L., Curto M.D.D., Loreti G., Gazzaniga A. (2010). Oral pulsatile delivery: Rationale and chronopharmaceutical formulations. Int. J. Pharm..

[3] Lopes C.M., Bettencourt C., Rossi A., Buttini F., Barata P. (2016). Overview on gastroretentive drug delivery systems for improving drug bioavailability. Int. J. Pharm..

[4] Rahamathulla M., Hani U., Alqahtani A., Gangadharappa H.V., Yasmin Begum M., Jafar M., Osmani R.A.M., Chidambaram K., Moin A., Shankar S.J. (2021). 2^3^ Factorial design and optimization of effervescent floating matrix tablet of neratinib. J. Pharm. Innov..

[5] Huh H.W., Na Y.G., Kang H.C., Kim M., Han M., Pham T.M.A., Lee H., Baek J.S., Lee H.K., Cho C.W. (2021). Novel self-floating tablet for enhanced oral bioavailability of metformin based on cellulose. Int. J. Pharm..

[6] Rahamathulla M., Alam M.D., Hani U., Ibrahim Q., Alhamhoom Y. (2021). Development and in vitro evaluation of effervescent floating matrix tablet of neritinib: An anticancer drug. Pak. J. Pharm. Sci..

[7] Eberle V.A., Schoelkopf J., Gane P.A.C., Alles R., Huwyler J., Puchkov M. (2014). Floating gastroretentive drug delivery systems: Comparison of experimental and simulated dissolution profiles and floatation behavior. Eur. J. Pharm. Sci..

[8] Teaima M., Hamid M.M.A., Shoman N.A., Jasti B.R., El-Nabarawi M.A. (2020). Promising swellable floating bupropion tablets: Formulation, in vitro/in vivo evaluation and comparative pharmacokinetic study in human volunteers. Drug Des. Dev. Ther..

[9] Chen N., Niu J., Li Q., Li J., Chen X., Ren Y., Wu G., Liu Y., Shi Y. (2019). Development and evaluation of a new gastroretentive drug delivery system: Nanomicelles-loaded floating mucoadhesive beads. J. Drug Deliv. Sci. Technol..

[10] Hwang K.M., Nguyen T.T., Seok S.H., Jo H.I., Cho C.H., Hwang K.M., Kim J.Y., Park C.W., Rhee Y.S., Park E.S. (2019). Swellable and porous bilayer tablet for gastroretentive drug delivery: Preparation and in vitro-in vivo evaluation. Int. J. Pharm..

[11] Atyabi F., Sharma H.L., Mohammad H.A.H., Fell T. (1996). Controlled drug release from coated floating ion exchange resin beads. J. Control. Release.

[12] Alladi K.K., Suram R., Bela M., Kiran S., Ramesh V., Narendera Y. (2011). Formulation and characterization of clarithromycin controlled release bioadhesive tablets. J. Chem. Pharm. Res..

[13] Rahamathulla M., Saisivam S., Gangadharappa H.V. (2019). Development of valsartan floating matrix tablets using low density polypropylene foam powder: In vitro and in vivo evaluation. AAPS PharmSciTech.

[14] Matharu A.S., Motto M.G., Patel M.R., Simonelli A.P., Dave R.H. (2011). Evaluation of hydroxypropyl methylcellulose matrix systems as swellable gastro-retentive drug delivery systems. J. Pharm. Sci..

[15] Cargill R., Caldwell L.J., Engle K., Fix J.A., Porter P.A., Gardner C.R. (1988). Controlled gastric emptying. Effects of physical properties on gastric residence times of nondisintegrating geometric shapes in beagle dogs. Pharm. Res..

[16] Kerdsakundee N., Mahattanadul S., Wiwattanapatapee R. (2015). Development and evaluation of gastroretentive raft forming systems incorporating curcumin-Eudragit^®^ EPO solid dispersions for gastric ulcer treatment. Eur. J. Pharm. Biopharm..

[17] Hani U., Osmani R.A.M., Alqahtani A., Ghazwani M., Rahamathulla M., Almordy S.A., Alsaleh H.A. (2021). 2^3^ Full factorial design for formulation and evaluation of floating oral in situ gelling system of piroxicam. J. Pharm. Innov..

[18] Moen M.D., Waqstaff A.J. (2005). Losartan: A review of its use in stroke risk reduction in patients with hypertension and left ventricular hypertrophy. Drugs.

[19] Saisivam S., Rahamathulla M., Shakeel F. (2013). Development of floating matrix tablets of losartan potassium: In vitro and in vivo evaluation. J. Drug Deliv. Sci. Technol..

[20] Gangadharappa H.V., Rahamath-Ulla M., Pramod-Kumar T.M., Shakeel F. (2010). Floating drug delivery system of verapamil hydrochloride using karaya gum and HPMC. Clin. Res. Regul. Aff..

[21] Sapavatu S.N., Jadi R.K. (2019). Formulation development and characterization of gastroretentive drug delivery systems of loratadine. Int. J. Appl. Pharm..

[22] Roy S.K., Das P., Mondal A., Mandal A., Kuotsu K. (2021). Design, formulation and evaluation of multiparticulate time programmed system of ramipril for pulsed release: An approach in the management of early morning surge in blood pressure. J. Drug Deliv. Sci. Technol..

[23] Barba A.A., Dalmoro A., Bochicchio S., Simone V.D., Caccavo D., Iannone M., Lamberti G. (2020). Engineering approaches for drug delivery systems production and characterization. Int. J. Pharm..

[24] Ulla M.R., Saisivam S. (2013). Floating matrix tablet of losartan potassium by using hydrophilic swelling polymer and natural gum. Turk. J. Pharm. Sci..

[25] Abou Youssef N.A.H., Kassem A.A., El-Massik M.A.E., Boraie N.A. (2015). Development of gastroretentive metronidazole floating raft system for targeting Helicobacter pylori. Int. J. Pharm..

[26] Raza A., Hayat U., Wang H.J., Wang J.Y. (2020). Preparation and evaluation of captopril loaded gastro-retentive zein based porous floating tablets. Int. J. Pharm..

[27] Shakeel F., Iqbal M., Ezzeldin E. (2016). Bioavailability enhancement and pharmacokinetic profile of an anticancer drug ibrutinib by self-nanoemulsifying drug delivery system. J. Pharm. Pharmacol..

[28] Note for Guidance on Stability Testing, Stability Testing of New Drug Substances and Products. http://www.ich.org/cache/compo/363-272-1.html.

[29] ICH Harmonized Tripartite Guidelines (2003). Stability testing of new drug substances and products. Q1A (R2). Fed. Reg..

[30] Srikanth M.V., Rao N.S., Sunil S.A., Ram B.J., Kolapalli V.R.M. (2012). Statistical design and evaluation of a propranolol HCl gastric floating tablet. Acta Pharm. Sin. B.

[31] Tadros M.I. (2010). Controlled-release effervescent floating matrix tablets of ciprofloxacin hydrochloride: Development, optimization and in vitro-in vivo evaluation in healthy human volunteers. Eur. J. Pharm. Biopharm..

[32] Saisivam S., Ulla M.R. (2011). Effect of sodium bicarbonate on the properties of losartan potassium floating tablet. Int. J. Pharm. Sci..

[33] Lin H.L., Chen L.C., Cheng W.T., Cheng W.J., Ho H.O., Sheu M.T. (2020). Preparation and characterization of a novel swellable and floating gastroretentive drug delivery system (sfGRDDS) for enhanced oral bioavailability of nilotinib. Pharmaceutics.

[34] Mamani P.L., Ruiz-Caro R., Veiga M.D. (2012). Matrix tablets: The effect of hydroxypropyl methylcellulose/anhydrous dibasic calcium phosphate ratio on the release rate of a water-soluble drug through the gastrointestinal tract I. In vitro tests. AAPS PharmSciTech.

[35] Prajapati P.H., Nakum V.V., Patel C.N. (2012). Formulation and evaluation of floating matrix tablet of stavudine. Int. J. Pharm. Investig..

[36] Streubel A., Siepmann J., Bodmeier R. (2003). Floating matrix tablets based on low density foam powder: Effects of formulation and processing parameters on drug release. Eur. J. Pharm. Sci..

